# A Path Planning Method with a Bidirectional Potential Field Probabilistic Step Size RRT for a Dual Manipulator

**DOI:** 10.3390/s23115172

**Published:** 2023-05-29

**Authors:** Youyu Liu, Wanbao Tao, Shunfang Li, Yi Li, Qijie Wang

**Affiliations:** 1 Anhui Key Laboratory of Detection Technology and Energy Saving Devices, Anhui Polytechnic University, Wuhu 241000, China; liuyyu@ahpu.edu.cn (Y.L.); 2210120152@stu.ahpu.edu.cn (W.T.); 2210110112@stu.ahpu.edu.cn (Y.L.); 2220110174@stu.ahpu.edu.cn (Q.W.); 2 Research Office, Wuhu Institute of Technology, Wuhu 241000, China; 3 Mechanical Engineering Department, Anhui Polytechnic University, Wuhu 241000, China

**Keywords:** dual manipulator, rapidly exploring random trees, angle selection, artificial potential field method, bidirectional potential field probabilistic step size, path optimization

## Abstract

The search efficiency of a rapidly exploring random tree (RRT) can be improved by introducing a high-probability goal bias strategy. In the case of multiple complex obstacles, the high-probability goal bias strategy with a fixed step size will fall into a local optimum, which reduces search efficiency. Herein, a bidirectional potential field probabilistic step size rapidly exploring random tree (BPFPS-RRT) was proposed for the path planning of a dual manipulator by introducing a search strategy of a step size with a target angle and random value. The artificial potential field method was introduced, combining the search features with the bidirectional goal bias and the concept of greedy path optimization. According to simulations, taking the main manipulator as an example, compared with goal bias RRT, variable step size RRT, and goal bias bidirectional RRT, the proposed algorithm reduces the search time by 23.53%, 15.45%, and 43.78% and decreases the path length by 19.35%, 18.83%, and 21.38%, respectively. Moreover, taking the slave manipulator as another example, the proposed algorithm reduces the search time by 6.71%, 1.49%, and 46.88% and decreases the path length by 19.88%, 19.39%, and 20.83%, respectively. The proposed algorithm can be adopted to effectively achieve path planning for the dual manipulator.

## 1. Introduction

Path planning is an essential component of robot motion planning and is a research hotspot in the field of robotics and other related intelligent fields [1]. Among them, the manipulator, as an important industrial robot, has autonomy and intelligence levels that are crucial for improving production efficiency and quality. Path planning can help the manipulator automatically plan the optimal path, reduce dependence on staff, and improve the autonomy of the manipulator. Path planning can be flexibly adjusted and optimized according to different task characteristics, thereby improving the motion accuracy and speed of the manipulator, which directly affects production efficiency and quality. Compared with a single manipulator, the form of a dual manipulator collaborative operation can meet the needs of complex, intelligent, and compliant modern industrial systems, and dual manipulators have more advantages in efficiency and performance [2,3] and are gradually gaining attention in the industry. Path planning for dual manipulators is an important work of collaborative operations [4]. To enhance the adaptability and flexibility of dual-manipulator systems, it is necessary to flexibly adjust and optimize paths based on various production environments and task requirements. The equipment transformation and upgrading of production lines are of great significance, but there are high requirements for efficiency, real-time performance, and safety in collaborative operations [5]. To address these challenges, researchers are continuously developing and improving various path planning methods for dual manipulators to enhance their efficiency and precision and meet different demands in industrial production environments. Therefore, different path planning algorithms need to be designed or selected for various fields to achieve the goal of fast matching and application.

## 2. Related Work

The classic 3D path planning algorithms for robots can be roughly divided into three categories. The first type of path planning algorithm is based on searches, such as Dijkstra and A* algorithms [6,7]. This algorithm is based on a graph structure in which each node represents the robot’s location, and each edge represents its movement path. By searching through the graph structure and calculating a heuristic function for each node to evaluate the distance to the endpoint, the optimal path is found. Qing et al. [8] proposed an improved Dijkstra algorithm that saves all equidistant shortest paths during the path search process, although it can solve several shortest path planning problems; in some cases, it may be difficult to obtain the complete graph structure, and there are issues such as large search space, high computational complexity, and poor real-time performance.

The second type of path planning algorithm is based on rules, such as the artificial potential field method [9,10]. The main idea is to design an artificial potential field to simulate the perception and decision-making process of the robot during movement and achieve path planning. The artificial potential field method has the advantages of simple algorithm implementation, easy understanding and use, rapid calculation of robot movement paths, and high real-time performance. Therefore, Xia et al. [11] proposed an improved velocity potential field (IVPF) algorithm based on the artificial potential field method to address the inherent drawbacks of traditional algorithms. However, utilizing tangential velocity to avoid local minimum problems leads to poor path quality. The artificial potential field method only considers the relationship between the robot and obstacles, ignoring the constraints among robots themselves, which may lead to locally optimal solutions in some cases.

To address this issue, the third type of path planning algorithm based on sampling is widely applied in various fields, such as the rapidly exploring random tree (RRT) [12] and probabilistic roadmap (PRM) [13]. The main idea is to search for the optimal feasible path through random sampling in the environment. Sampling-based algorithms are not limited by the type of environment and can be applied to path planning problems in various complex environments, with high robustness and reliability. Li et al. [14] improved the PRM algorithm by using a pseudorandom sampling strategy with the spatial principal axis as a reference axis and optimized the path using Bezier curves. However, the roadmap construction rate is unstable in three-dimensional environments. Liu et al. [15] proposed a grid-local PRM method, which has high efficiency and real-time performance. However, this type of algorithm has weak scalability and a low roadmap reuse rate. To address this issue, the RRT algorithm and its variations have been proposed. The RRT algorithm has wide applicability, high efficiency, strong scalability, good determinism, and real-time computation, which effectively solves the path planning problem with high-dimensional space constraints. As a result, the RRT algorithm has become one of the most commonly used and effective algorithms in path planning.

On this basis, Kuffner et al. [16] proposed an RRT-connect double tree algorithm by randomly expanding paths at the same time at the start and goal nodes. It is superior to the RRT algorithm in terms of search performance. However, it is difficult to find the optimal path due to randomness. To solve this problem, scholars made some improvements to the RRT-connect algorithm. For example, based on the triangle inequality, Kang et al. [17] proposed an RRT-connect algorithm based on the triangle inequality principle by re-wiring path nodes, which has outstanding performance in terms of path length. However, there may be problems, such as non-differentiable linear sections with sharp corners and constraints with the kinematics of the manipulator. Based on the idea of dynamic step size [18], Li et al. [19] proposed a variable step size RRT (VT-RRT) by transforming the search space of random nodes in the RRT algorithm and adaptively adjusting the search step size according to the goal and the position of obstacles in the current point. This algorithm effectively reduces path planning time and optimizes sampling direction. However, it generates too many path nodes, resulting in longer paths. To improve the adverse effects of variable step size, Zhang et al. [20] proposed a path planning method for a manipulator based on the artificial potential field and bidirectional rapidly exploring random tree (BiRRT-APF) algorithm, aiming to solve the problem of low search efficiency and high randomness. However, its goal orientation is poor. Shao et al. [21] proposed a motion planning method based on the goal bias RRT algorithm (G-RRT), which reduces invalid searches by guiding the direction of random sampling. However, the one-way search is less efficient, and the resulting path is not optimal. Liu et al. [22] proposed a goal bias bidirectional rapidly exploring random tree (GBI-RRT) algorithm, which improves the success rate of node expansion. However, in complex and high-dimensional environments, this algorithm generates redundant nodes, resulting in overly complex paths. The types of path planning algorithms are shown in Table 1 below, as well as the advantages and disadvantages of each algorithm. Due to the existence of overlapping workspaces, the path planning of dual manipulators should deal with the interference of static and dynamic obstacles at the same time. In response to the above content, a sampling-based RRT path planning algorithm is adopted to improve and optimize the shortcomings of the algorithm and is deployed on a dual manipulator.

Regarding the aforementioned issues, this paper proposes a bidirectional potential field probabilistic step-size RRT algorithm for the path planning of dual manipulators by angle selection. The main contributions of this paper are as follows:

(1) Based on the RRT-connect algorithm and the characteristics of bidirectional searches, the high goal probability bias strategy is introduced to enable the random points to be sampled along the goal direction.
(2) Angle selection is used to limit the direction of dual-tree searches and avoid redundant sampling to the surrounding area.
(3) Based on the idea of dynamic step size, random values are innovatively used as step size parameters, and the search step size is adaptively adjusted by the dynamic changes of randomness to cope with the environment. The artificial potential field method is introduced to deal with multi-obstacle environments.
(4) A greedy algorithm is used for path optimization, removing redundant nodes on the path and finding the shortest path.

The remainder of this article is organized as follows. Section 3 introduces the posture description and collision detection of the dual manipulator. Section 4 presents the improved strategies and algorithm flow of the proposed algorithm. In Section 5, comparative experiments are conducted with RRT-Goalbias, VT-RRT, and goal-biased bidirectional RRT as the control groups, and an ablation experiment is also carried out on the proposed algorithm to validate its performance. Section 6 provides a path planning case study, deploying the proposed algorithm for obstacle avoidance path planning on a dual-arm robot for simulation verification. Section 7 outlines limitations and future scope, and Section 8 presents conclusions.

## 3. Posture Description and Collision Detection

### 3.1. Posture Description

**Figure 1:**
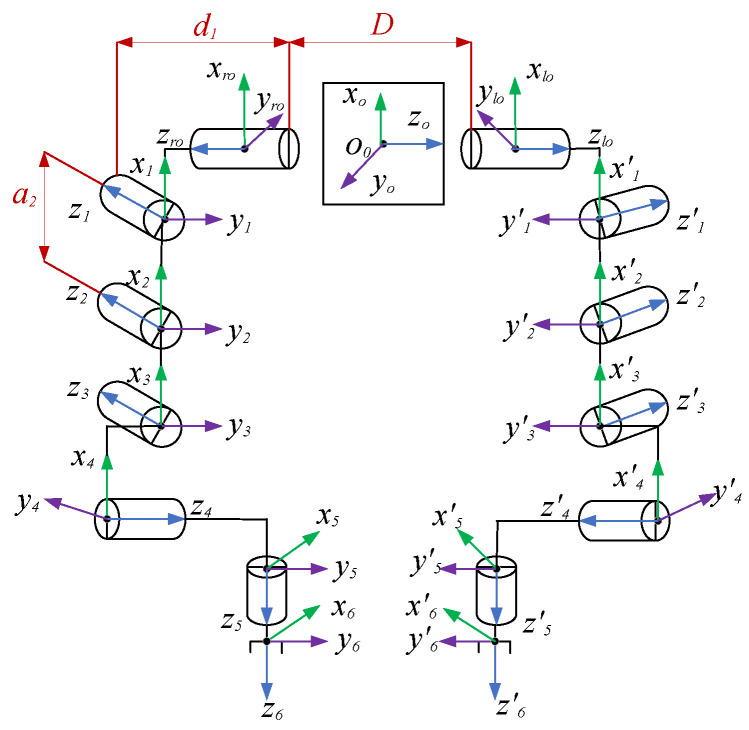
Structure diagram of the dual manipulator.

The dual manipulator model is composed of the double PUMA560 manipulators, and the DH modeling method [23] is used to establish the link coordinate system of the dual manipulator. As shown in Figure 1, ($x_{o},y_{o},z_{o}$) is the global base coordinate system, ($x_{lo},y_{lo},z_{lo}$) and ($x_{ro},y_{ro},z_{ro}$) are the base coordinate systems of the left and right manipulators, respectively, and ($x_{i},y_{i},z_{i}$) and ($x_{i}^{\prime },y_{i}^{\prime },z_{i}^{\prime }$) are the coordinate systems of the left and right manipulator joints i, respectively.

Table 2 presents the DH parameters of the main manipulator; the mirror image of the main and slave manipulators is symmetrical and α and θ are opposite to each other, where D = 1 m, $d_{3}$ = 0.1500 m, $d_{4}$ = 0.4318 m, $a_{2}$ = 0.4318 m, and $a_{3}$ = 0.0203 m. The DH parameters of a rigid link depend on the link parameters of the member (i.e., $a_{i}$ and $\alpha_{i}$) and the joint parameters of the adjacent links (i.e., $d_{i}$ and $\theta_{i}$).

The two-link offset $d_{i}$ is the moving joint variable and the joint angle of the two links $\theta_{i}$ is the variable of rotating joints. The pose of the adjacent links is expressed by the $T_{i}^{i-1}$ matrix [24] as:

(1) $$T_{i}^{i-1}=R_{rot}\left(z_{i-1},\theta_{i}\right){\cdot T}_{trans}\left(z_{i-1},d_{i}\right)\cdot T_{trans}\left(x_{i-1},a_{i}\right){\cdot R}_{rot}\left(x_{i-1},\alpha_{i}\right)$$

Equation (2) shows the main manipulator homogeneous coordinate transformation matrix.

(2) $$T_{i}^{i-1}=\left[\begin{array}{c} c_{\theta_{i}}\\ s_{\theta_{i}}\\ 0\\ 0 \end{array}\begin{array}{c} {-s}_{\theta_{i}}c_{\alpha_{i}}\\ c_{\theta_{i}}c_{\alpha_{i}}\\ s_{\alpha_{i}}\\ 0 \end{array}\begin{array}{c} s_{\theta_{i}}s_{\alpha_{i}}\\ {-c}_{\theta_{i}}s_{\alpha_{i}}\\ c_{\alpha_{i}}\\ 0 \end{array}\begin{array}{c} a_{i}c_{\theta_{i}}\\ a_{i}s_{\theta_{i}}\\ d_{i}\\ 1 \end{array}\right]$$

Substituting the DH parameter, the transformation matrix [25] relative to the base coordinate system is shown in Equation (3):

(3) $${T=T}_{1}^{0}T_{2}^{1}T_{3}^{2}T_{4}^{3}T_{5}^{4}T_{6}^{5}$$

The transformation matrix of the main manipulator is unified in the base coordinate system [26] by Equation (3), as shown in Equation (4):

(4) $$S=S_{base}T$$

The slave manipulator pose is also calculated according to the above steps.

### 3.2. Collision Detection

**Figure 2:**
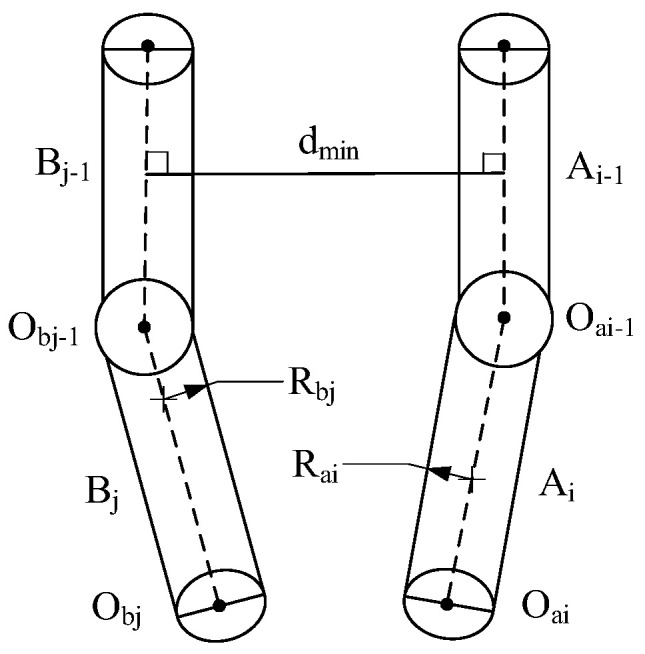
A dual manipulator bounding box model.

The hemispherical–cylindrical enveloping box method [27] is employed. Figure 2 shows the proposed dual manipulator encirclement box model. The collision detection mainly judges the distance between the $d_{min}$ and $\left(R_{ai}+R_{bj}\right)$. The dual manipulator overlapping workspace is obtained based on the Monte Carlo method [28]. Equations (5)–(7) show the collision conditions [29].

If the two vertical feet are inside the links, the shortest distance $d_{min}$ is as follows:

(5) $$d_{min}=min\left\{{\left\|A_{i}B_{j}\right\|}_{2}\right\}$$

If one of the vertical feet is on the extension of the links, then the shortest distance $d_{min}$ is as follows:

(6) $$d_{min}=min\left\{{\left\|A_{i}O_{bj}\right\|}_{2},{\left\|B_{j}O_{ai}\right\|}_{2}\right\}$$

If both the vertical feet are outside the extension line of the links, the shortest distance $d_{min}$ is as follows:

(7) $$d_{min}=min\left\{{\left\|O_{ai}O_{bj}\right\|}_{2}\right\}$$

where i=1,2,3⋯6,j=1,2,3⋯6. If $d_{min}>R_{ai}+R_{bj}$, the links of the main slave manipulators do not collide. Conversely, a collision occurs between the links.

## 4. Motion Planning

### 4.1. Improvement of the RRT Algorithm

#### 4.1.1. RRT-Connect Algorithm

**Figure 3:**
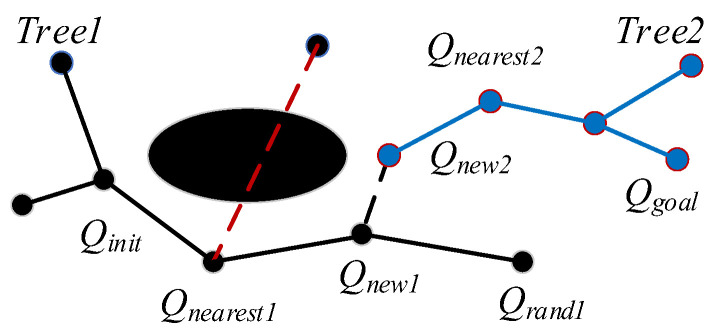
A schematic diagram of double tree expansion.

This algorithm defines two random trees, namely, Tree1 and Tree2. Tree1 is expanded from the starting point $Q_{init}$ and Tree2 is expanded from the goal point $Q_{goal}$. Sampling generates random nodes $Q_{rand}$, the existing random trees are traversed, and $Q_{nearest}$ is found to be the closest to $Q_{rand}$ from the node in the tree. $Q_{nearest}$ extends to $Q_{rand}$ by step size δ and obtains the new node $Q_{new}$. The collision detection is performed on $Q_{new}$. If $Q_{new}$ hits an obstacle, the node is dropped; otherwise, $Q_{new}$ is added to the tree. At this time, the parent node of $Q_{new}$ is $Q_{nearest}$ and continues to expand according to the above way, until the $Q_{new}$ of the two trees is less than the step size threshold. Tree1 and Tree2 can then be connected, that is, the path planning is successful. Figure 3 shows the growth process of the random tree of the RRT-connect.

#### 4.1.2. Strategy for Step Size Selection

The traditional RRT algorithm has a large number of blind searches, which reduces the convergence speed and occupies a lot of computational power. To solve this problem, a high-probability goal bias strategy [30] is used to guide the random tree to search in the direction of the goal with probability. The sampling direction is derived from Equation (8):

(8) $$Direction=\left\{\begin{array}{c} goal,\;0<\gamma <Pa\\ rand,\;Pa\leq \gamma \leq 1\\ \gamma \in \left[0,1\right] \end{array}\right.$$

By employing this strategy, the probability of sampling pointing toward the goal direction is increased. In addition, a bidirectional potential field probabilistic step size strategy based on angle selection is proposed. Unlike conventional variable step size, the algorithm instead utilizes a random value γ as the variable step size parameter. The starting point and the goal point search for each other by introducing the goal bias probability. The step size amount of the goal bias is increased, thereby improving the algorithm’s efficiency.

The process configuration of RRT-connect [31] grows the new node $Q_{new}$, as shown in Equation (9):

(9) $$Q_{new}=Q_{nearest}+\delta \frac{Q_{nearest}-Q_{rand}}{\left|Q_{nearest}-Q_{rand}\right|}+\delta \frac{Q_{nearest}-Q_{goal}}{\left|Q_{nearest}-Q_{goal}\right|}$$

The goal bias and probabilistic step size $\delta_{1-2}$ are introduced, and the new node $Q_{new1}$ expansion direction of the starting point [31] is shown in Equation (10):

(10) $$Q_{new1}=Q_{nearest1}+\delta_{1}\frac{Q_{nearest1}Q_{rand1}}{{\left\|Q_{nearest1}-Q_{rand1}\right\|}^{2}}+\delta_{2}\frac{Q_{nearest1}Q_{goal}}{{\left\|Q_{nearest1}-Q_{goal}\right\|}^{2}}$$

The new node $Q_{new2}$ expansion direction of the goal point is given by Equation (11):

(11) $$Q_{new2}=Q_{nearest2}+\delta_{1}\frac{Q_{nearest2}Q_{rand2}}{{\left\|Q_{nearest2}-Q_{rand2}\right\|}^{2}}+\delta_{2}\frac{Q_{nearest2}Q_{init}}{{\left\|Q_{nearest2}-Q_{init}\right\|}^{2}}$$

**Figure 4:**
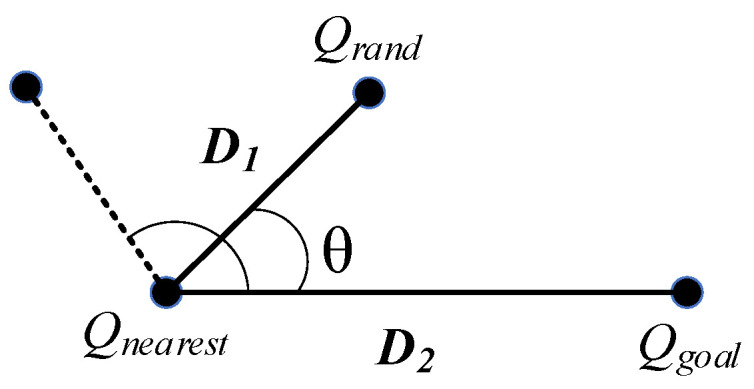
A diagram of angle selection.

Figure 4 shows the angle selection of $Q_{rand1}$ and $Q_{rand2}$. Equation (12) shows the vector representations of random points and goal points [32].

(12) $$\left\{\begin{array}{c} D_{1}=Q_{rand}-Q_{nearest}\\ D_{2}=Q_{goal}-Q_{nearest} \end{array}\right.$$

The angle expression is given by Equation (13):

(13) $$\theta =\left|arccos(\frac{D_{1}\cdot D_{2}}{\left|D_{1}\right|\cdot \left|D_{2}\right|})\right|$$

To improve the search capability for complex environments, the step size of the angle selection expression is shown in Equation (14):

(14) $$S=\left\{\begin{array}{c} \;\delta_{1}=\delta \cdot \gamma ,\;\theta \epsilon (\frac{\pi }{2},\pi ]\\ \delta_{2}=\delta \cdot \left(2-\gamma \right),\;\theta \epsilon \left[0\right.,\left.\frac{\pi }{2}\right] \end{array}\right.$$

where both $\delta_{1}$ and $\delta_{2}$ step sizes are randomly varied. When 0≤θ≤π/2, the random point sampling direction is biased toward the goal, and the random value is used as the step size growth rate to expand toward the goal point; when π/2<θ≤π, the random point sampling direction deviates from the goal, and the random value is used as the step size magnification to deviate from the goal point expansion. Due to the bias of sampling points toward the target, with γ between 0 and Pa, using γ as a step growth rate provides only a small growth advantage. Instead, (1−γ) is used as the step growth rate.

The artificial potential field method is introduced to improve the step size step efficiency by integrating the potential field method. The gravitational field step size expression [33] is shown in Equation (15):

(15) $$F_{g}=S\cdot k_{g}\frac{Q_{nearest}Q_{goal}}{{\left\|Q_{nearest}-Q_{goal}\right\|}^{2}}$$

Additionally, the step size of the repulsive field [33] is expressed as:

(16) $$F_{r}=S\cdot k_{r}\frac{Q_{nearest}Q_{rand}}{{\left\|Q_{nearest}-Q_{rand}\right\|}^{2}}$$

Additionally, the step size of the combined force is shown in Equation (17):

(17) $$F={F_{g}+F}_{r}$$

**Figure 5:**
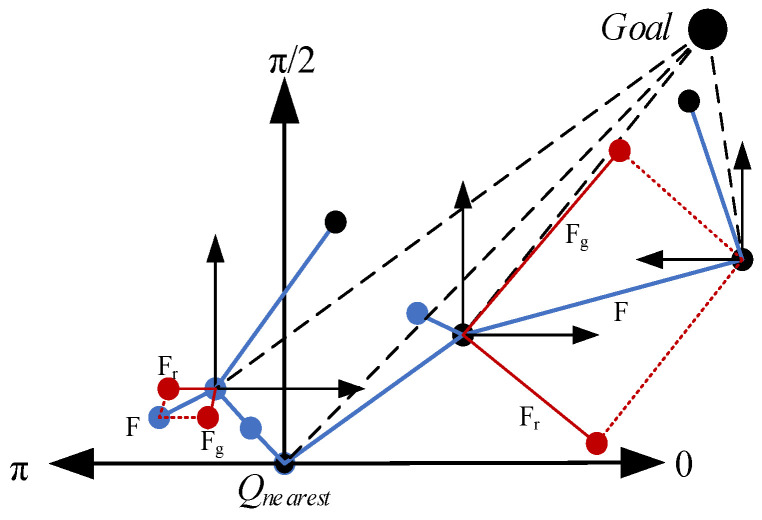
Improving the sampling process with step size.

In the improved step size sampling process illustrated in Figure 5, if the directions of $Q_{nearest}$ and the goal fall within the range of 0 to π/2 (indicated by black dots in the figure), the step sizes all follow the growth of $\delta_{2}$. After incorporating the potential field method, the step sizes increase with length F, including the gravitational step size $F_{g}$ and repulsive step size $F_{r}$ (indicated by the red dot in the figure). Conversely, when moving away from the goal, the step sizes all follow the growth of $\delta_{1}$ (indicated by blue dots in the figure). This results in a shortening of the length of F. By taking small and variable step sizes, it becomes easier for the algorithm to adapt to complex environments. The random value is used as the step size parameter to maintain the randomness of the sampling algorithm and accelerate the convergence speed.

#### 4.1.3. Greedy Algorithm for Path Optimization

The fixed step size produces redundant path nodes. A greedy algorithm [34] is used to remove redundant nodes. Therefore, the optimal path at the moment is constructed. The path nodes are obtained by the proposed algorithm to define the nodes set C∈[S,N], as shown in Equation (18):

(18) $$N=({n_{1},\;n_{2},\;n_{3},\;\cdots ,\;n}_{i-1},\;n_{i})$$

where i=2,3,4⋯. 

The mathematical model of the traversing process [35] is shown in Equation (19):

(19) $$OPT\left(S,G\right)=\left\{\begin{array}{c} OPT(S,n_{i-1})\\ check\{OPT\left(S,n_{i}\right)\} \end{array}\right.$$

S is set as a fixed point. If there is an obstacle between S and $n_{i}$, the retention point v is returned, as shown in Equation (20):

(20) $$OPT\left(v,G\right)=\left\{\begin{array}{c} OPT(v,n_{j-1})\\ check\{OPT\left(v,n_{j}\right)\} \end{array}\right.$$

where j=i−1. 

The iterative endpoint expression is shown in Equation (21).

(21) $$OPT\left(v,n_{j}\right)=check\{OPT\left(v,G\right)\}$$

In i−1 iterations, the v of each round is added to the set, and the path optimization is completed. The path is shown in Equation (22):

(22) $$S\to n_{i-1}\to \cdots \to n_{j-1}\to G$$

**Figure 6:**
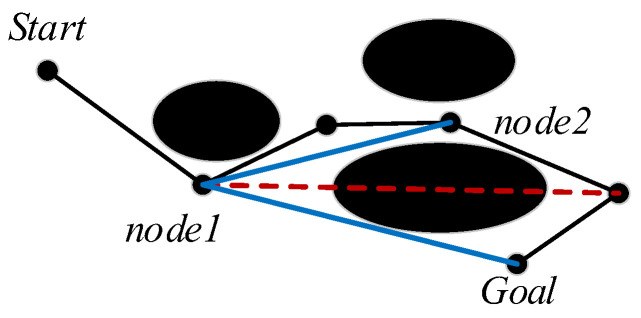
A schematic diagram of path optimization.

The greedy algorithm is used to traverse all the nodes along the path, and all the redundant nodes are checked and deleted, as shown in Figure 6, where the black route, the red dashed line, and the blue line represent the original path, the collision path, and the optimization path, respectively. In the optimization path Start→node1→node2, it has one redundant node and deletes it. When traversing to the goal point Goal, it has three redundant nodes and deletes them, and then the optimal path is Start→node1→Goal.

### 4.2. Process of the BPFPS-RRT Algorithm

In the proposed BPFPS-RRT algorithm, the input consists of two random trees, namely, Tree1 and Tree2, and the output is the optimization path, as shown in Algorithm 1. First, a point $Q_{rand}$ is sampled, and the start and goal nodes are then expanded in the direction of $Q_{rand}$ at the same time. Second, the new nodes $Q_{new1}$ and $Q_{new2}$ are obtained by the extending algorithm Extend(T,Q) (lines 4–8). If the distance between $Q_{new1}$ and $Q_{new2}$ is less than the step threshold, then the cycle is terminated, and the path between Tree1 and Tree2 is connected. Finally, the greedy algorithm is used to generate the optimal path (lines 9–16).
**Algorithm 1:** Pseudocode for BPFPS-RRT**Input:**The two random trees, Tree1 and Tree2.**Output:**Connection from T_1_ to T_2_, path optimization.1:*T* = initialize (Tree1, Tree2);2:δ = initialize ();3:T [0] = Node ($Q_{init}$, $Q_{goal}$);4:**for**  i = 1 to M **do**5:  $Q_{rand}$ ← Sample(random);6:  *Q_new1_*
← Extend (Tree1, $Q_{goal}$);7:  **if**
*Q_new1_* ≠ NULL, **then**8:    *Q_new2_* ← Extend (Tree2, $Q_{init}$)9:    **if**
*Distance* (*Q_new1_*, *Q_new2_*) < δ, **then**10:     return path (Tree1, Tree2)11:    **end if**12:  **end if**13:  path optimization ← *OPT* (path);14:  return path optimization15:  swap (Tree1, Tree2)16:**end for**

Algorithm 2 shows the pseudocode for the Extend(T,Q) algorithm. The input is the high goal probability threshold Pa, the random value γ, and step size δ, and the output is the new node $Q_{new1}$. First, if the random value is greater than the threshold Pa, then $Q_{rand}$ is randomly sampled. Conversely, $Q_{rand}$ samples to the goal point and calculates the angle θ between $D_{1}$ and $D_{1}$ (lines 1–4). Second, the range of angle θ is evaluated using the conditional statement. Based on the result of this evaluation, the suitable step size for the search is determined. The artificial potential field method is then incorporated to compute the combined force and obtain $Q_{new}$ (lines 5–10). Finally, the *CollisionFree* algorithm is used to determine the path between $Q_{nearest}$ and $Q_{new}$. If the path exists, then $Q_{new}$ is added to the node set of the random tree, and edges ($Q_{nearest}$, $Q_{new}$) are added to the edge set of the random tree (lines 11–14).
**Algorithm 2:** Pseudocode for extend (*T, Q*)**Input:**Goal-bias Pa. Random value γ. The step size δ.**Output:**$\mathrm{the}\;\mathrm{new}\;\mathrm{node},\;Q_{new}$1:**if** γ > *P_a_*2:$Q_{rand}$← Sample(random); else3:$Q_{rand}$← Sample(goal); **end if**4:θ$=|\;\mathrm{cal}\;(D_{1}$*_,_*$D_{2}$) |;5:if  0≤θ≤π/26:    $\mathrm{step}\;\mathrm{size}=\delta \cdot \left(2-\gamma \right)$; else7:    step size=δ·γ;8:**end if**9:$F={F_{g}+F}_{r}$10:$Q_{new}$$=Q_{nearest}+F$11:**if** *CollisionFree* $(Q_{nearest}$$,\;Q_{new}$), **then**12:    add-vertex $\;(Q_{new}$), add-edge $(Q_{nearest}$$,\;Q_{new}$); 13:**end if**14:$\mathrm{return}\;Q_{new}$

## 5. Planning Simulation

### 5.1. Performance of the BPFPS-RRT Algorithm

The simulation is performed using MATLAB 2020b. The hardware is an Intel (R) Core (TM) i7-12700H CPU. The reference frequency is 2.70 GHz and the memory is 16 G. The initial conditions of the simulation are as follows: the starting point coordinates are [10, 10, 10], the endpoint coordinates are [150, 150, 150], and the initial step size is δ = 10 mm, $k_{g}$ = 1.5, and $k_{r}$ = 1. The five balls of different specifications are simulated as obstacles in a three-dimensional space, and the G-RRT, VT-RRT, and GBI-RRT are selected as the control groups.

**Figure 7:**
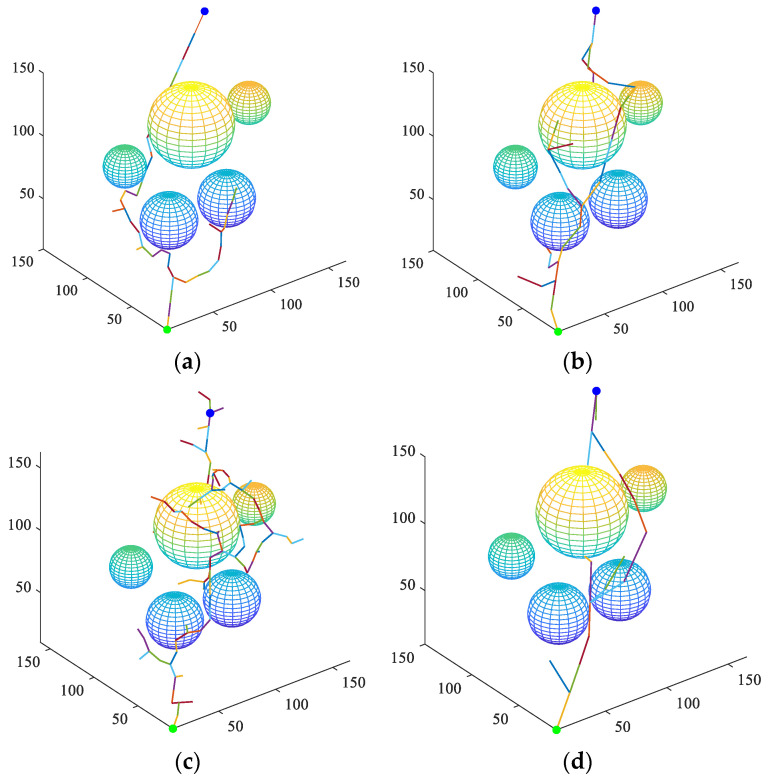
The search process of the four algorithms. (**a**) G-RRT, (**b**) VT-RRT, (**c**) GBI-RRT, and (**d**) BPFPS-RRT.

Figure 7 shows the search process of the control groups and the proposed BPFPS-RRT. Figure 7a–d shows the search path process of the G-RRT, VT-RRT, GBI-RRT, and BPFPS-RRT algorithms, respectively. The green and blue dots represent the starting point and the goal point, respectively. The control groups are compared to the BPFPS-RRT. As shown in Figure 7a, the G-RRT produces redundant sampling in the surrounding environment. As shown in Figure 7b, the two trees use variable step size sampling, but their tendency toward the goal is poor. As shown in Figure 7c, the two nodes expand toward each other; however, a large number of tree nodes are generated during the sampling process, and the fixed step size produces a complex path. As shown in Figure 7d, the proposed algorithm has fewer path nodes, and the search step size can be adaptively adjusted based on the surrounding environment. This method avoids the failure of local planning and improves the efficiency of the path search.

**Figure 8:**
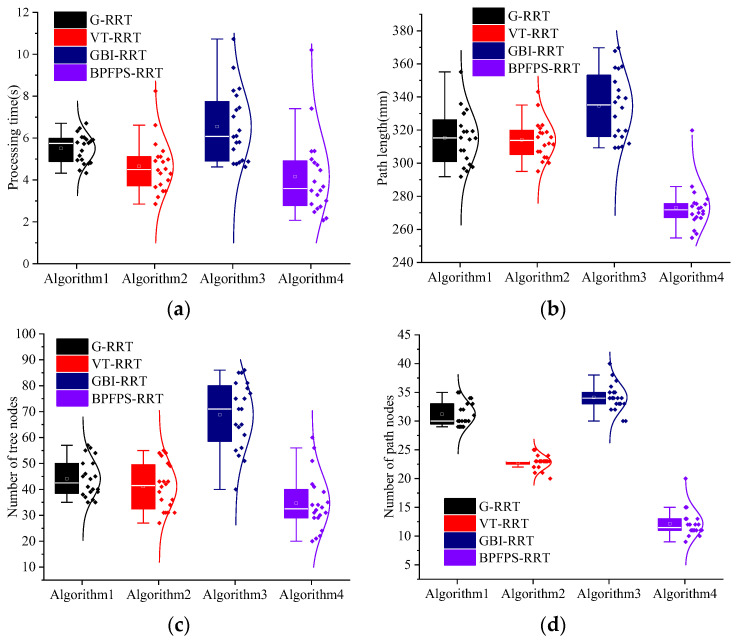
Simulation data of the four algorithms. (**a**) Search time, (**b**) path length, (**c**) number of tree nodes, and (**d**) number of path nodes.

The experiment is repeated 20 times to explore the algorithm search time and planning path performance. Figure 8a–d shows the search time, path length, tree nodes, and number of path nodes of the four algorithms, respectively. The data differences of each algorithm are visually reflected by the box plot and normal curve. Table 3 presents the average experimental data of the four algorithms.

Compared to the G-RRT algorithm, the search time is reduced by 24.50% and the number of path nodes is reduced by 58.06%. Because the G-RRT searches in one direction, it is easy to increase the number of path nodes in complex environments. Among them, compared to the G-RRT algorithm, the path length of the BPFPS-RRT algorithm is shortened by 13.38%, and the number of tree nodes is reduced by 20.45%. These results suggest that BPFPS-RRT generates fewer nodes, makes better use of path points, and exhibits better overall performance compared to G-RRT.

The search time of the BPFPS-RRT is 10.76% shorter than the VT-RRT, and the number of tree nodes and path length of the BPFPS-RRT are reduced by 14.63% and 13.03%, respectively. The variable step size of the VT-RRT performs better than the G-RRT and GBI-RRT in complex environments but lacks potential field guidance, and its overall performance is inferior to the BPFPS-RRT. The number of path nodes was reduced by 43.48% compared with the VT-RRT. Based on data comparison, it is found that there are too many redundant nodes present in the vicinity and the path utilization rate is also low.

Compared to the GBI-RRT algorithm, the search time of the BPFPS-RRT is reduced by 36.39%, the path length is shortened by 18.38%, the number of tree nodes is reduced by 48.53%, and the number of path nodes is reduced by 61.76%. Since the fixed step size of the GBI-RRT can easily fall into a dead corner in a complex environment, it jumps out of the local planning by increasing the number of tree nodes. These findings indicate that in complex environments, the BPFPS-RRT exhibits a significant reduction in extension nodes. There is a stronger tendency toward the search goal in its sampling process, the path length is shorter, and it takes less time.

### 5.2. Ablation Experiment

**Figure 9:**
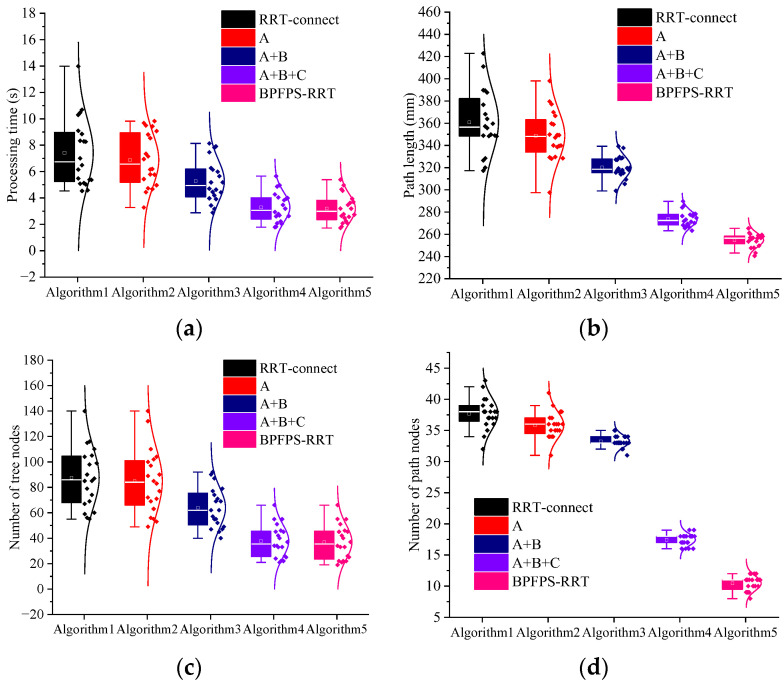
Simulation data of the five algorithms. (**a**) Search time, (**b**) path length, (**c**) number of tree nodes, and (**d**) number of path nodes.

To further visualize the improvement effects of each optimization strategy, an ablation experiment is conducted. The four main contribution strategies in Section 2 are, respectively, designated algorithm A, algorithm B, algorithm C, and algorithm D. The basic algorithm is RRT-connect, and the algorithm (A + B + C + D) is called the BPFPS-RRT algorithm. The simulation uses the same specific parameters as above and simulates obstacles in a three-dimensional space using four different sizes of spheres. The coordinates of their centers are (100, 100, 100), (50, 60, 60), (100, 60, 60), and (50, 110, 80), with radii of (30, 20, 20, 20), respectively. The experiment is repeated twenty times, simulation data for five algorithms as shown in Figure 9, and average experimental data for the five algorithms, as shown in Table 4, are obtained.

Through box plots and normal distribution curves, it is intuitively reflected that the performance is improved with the addition of each optimization strategy. Combining Figure 9 and Table 4 for analysis, algorithm A introduces a high goal probability bias strategy based on RRT-connect, so it improves by 7.28%, 3.35%, 3.41%, and 5.26% in terms of search time, path length, tree nodes, and path nodes compared to RRT-connect, respectively. By increasing the randomness in the search toward the goal direction, although the improvement effect is relatively small, it can provide assistance for subsequent improvements. Algorithm (A + B) adds a strategy that restricts the dual-tree search direction using angle selection, compared to algorithm A. In terms of performance, algorithm (A + B) improves by 23.40%, 8.20%, 24.71%, and 8.33% compared to algorithm A, respectively. It can be observed that there is a significant improvement in search time and tree nodes because the efficiency of searching toward the goal point is further improved by restricting the search direction, and redundant sampling of the surrounding environment is avoided as much as possible. Algorithm (A + B + C) adds a potential field probability step size compared to algorithm (A + B). In terms of performance, algorithm (A + B + C) improves by 37.76%, 14.48%, 40.63%, and 48.48% compared to algorithm (A + B), respectively. The random value is used as the step parameter, which changes with the variation of high goal probability bias, and through the guidance of the artificial potential field method, it can adaptively adjust the search step length. From the analysis of the number of nodes in the algorithm, the improvement is significant, with a reduction of approximately 20 nodes. The reduction in tree and path nodes indicates an improvement in the sampling efficiency of the goal. The search time has been improved by 2 s, and the path length has been significantly improved compared to the previous groups, with a length reduction of nearly 50 mm; this has resulted in a qualitative improvement in overall performance. The BPFPS-RRT adds a path optimization strategy compared to algorithm (A + B + C). In terms of performance, the BPFPS-RRT improves by 2.74%, 6.98%, 7.89%, and 41.18% compared to algorithm (A + B + C), respectively. The greedy algorithm optimizes the path mainly by trimming the path nodes and removing redundant nodes so that the path quality is improved. There is a significant improvement in the performance of the path nodes, with the optimized path length reduced by approximately 20 mm. The search time is improved by about 0.09 s, which can be ignored as it is an optimization of the path and has no effect on the search efficiency and falls within the normal fluctuation range.

Through the ablation experiment, the simulation experiments are conducted for each improvement strategy point, and it can be seen that among the five sets of simulation data comparisons, the performance improvement of the algorithm after adding the potential field probability step size is the largest, which is also the most important improvement point in this paper.

## 6. Path Planning Case

The three-dimensional obstacle environment and the dual manipulator model are set up to verify the effectiveness of the obstacle avoidance path planning of the dual manipulator. The collision detection model of the boundary ball is adopted. Four spherical static obstacles with a radius of 0.1 cm were established, and their center locations, along with the motion parameters of the manipulator, are presented in Table 5. In this section, the units of the parameters δ, $F_{g}$, $F_{r}$, and F are all in centimeters.

**Figure 10:**
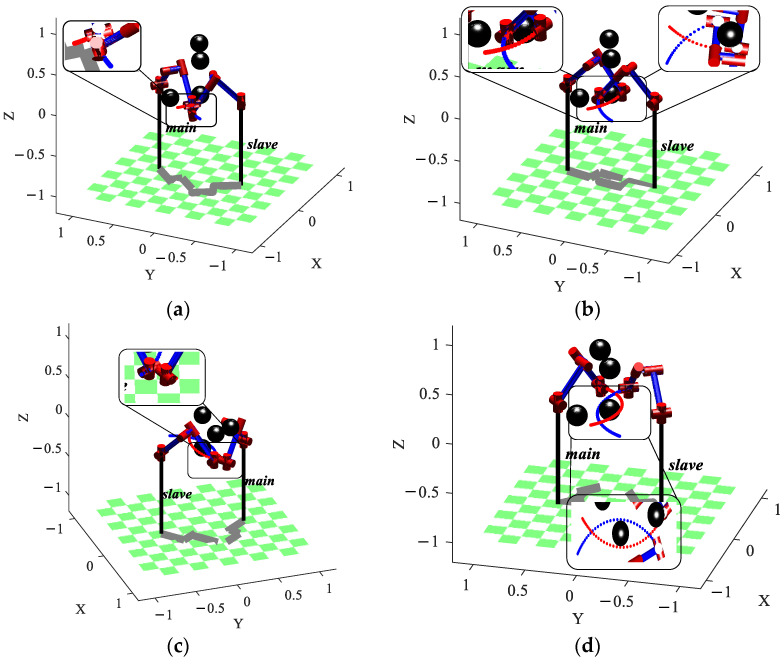
Dual manipulator pathless planning scenario, (**a**,**c**) dual manipulator collision, (**b**) collision with obstacles, (**d**) final state of movement.

**Figure 11:**
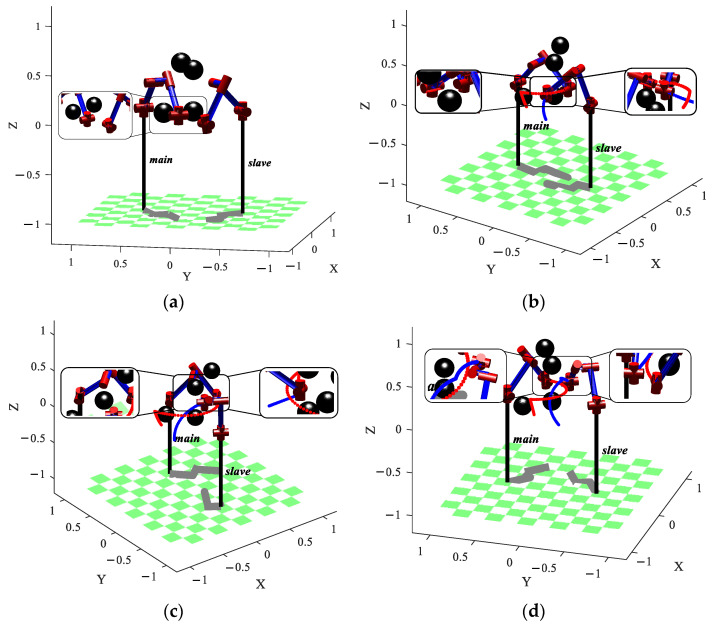
Movement process of the dual manipulator. (**a**) The initial state of movement, (**b**,**c**) obstacle avoidance process, (**d**) final state of movement.

The obstacles are placed on the path of the dual manipulator moving toward the goal point. Figure 10 shows the dual manipulator pathless planning scenario, where the red and blue colors represent the obstacle avoidance trajectory of the main manipulator and the obstacle avoid-ance trajectory of the slave manipulator, respec-tively. As depicted in Figure 10a,c, in the ab-sence of an obstacle avoidance path, the slave manipulator collides with the main manipulator during the dual manipulator’s motion. As shown in Figure 10b, the dual manipulator col-lides with static obstacles, and the final motion trajectory without path planning is shown in Figure 10d. Throughout the entire motion pro-cess, the slave manipulator failed to avoid ob-stacles and the moving main manipulator. While the environment around the dual manip-ulator is unchanged, the BPFPS-RRT path plan-ning algorithm is deployed in the simulation. Figure 11 shows the motion process of the dual manipulator, Figure 11a shows the initial movement of the dual manipulator, and both are without contact with obstacles. Figure 11c,d show the obstacle avoidance process of the dual manipulator. It is observed that the dual ma-nipulator avoids obstacles, and the slave ma-nipulator avoids not only obstacles but also the moving main manipulator without collision. Figure 11d shows the final state of movement of the dual manipulator.

**Figure 12:**
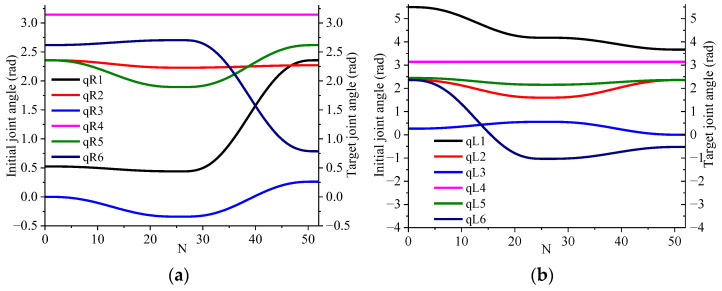
Dual manipulator joint angle change. (**a**) Main manipulator, (**b**) slave manipulator.

Figure 12 depicts the change in joint angles in the dual manipulator, where the sampling interval is represented by the abscissa N. As shown in Figure 12a, the obstacle avoidance process during the main manipulator’s motion from the starting point to the goal point is somewhat complex, but the degree of completion is high. The joint angles exhibit significant fluctuations in the middle of the movement, but the overall trajectory is smooth, and the main manipulator reaches the target position successfully. The joint angle change in the slave manipulator is presented in Figure 12b. The path planning from the starting point to the goal point is executed successfully. During the motion process, the slave manipulator primarily adheres to the obstacle avoidance requirements of the main manipulator, and the joint angle changes remain stable, ultimately successfully reaching the goal position.

These algorithms are applied to the dual manipulator simulation. In the event that the path produced by the algorithm is excessively lengthy or intricate, it may lead to singular configurations of the dual manipulator while executing the motion and, therefore, be interpreted as a failure of the path planning strategy. Table 6 presents the average operation data results after 20 sets of path planning experiments. For the main manipulator, the BPFPS-RRT is compared with the control group method, the path length of the BPFPS-RRT is reduced by 19.35%, 18.83%, and 21.38%. The path length of the control group method is close. The search time of the BPFPS-RRT is reduced by 23.53%, 15.45%, and 43.78%. When compared with the BPFPS-RRT, GBI-RRT requires more time due to its inclination to get trapped in local planning, which increases the search time. Although the path length of the GBI-RRT is similar to other control groups, its overall superiority is not as pronounced as that of the proposed algorithm. This characteristic can be attributed to the adaptive variation of its potential field probabilistic step size, which helps avoid a substantial number of redundant tree nodes. For the slave manipulator, the search time of the BPFPS-RRT is reduced by 6.71%, 1.49%, and 46.88% compared to the control groups, respectively. The path lengths of the BPFPS-RRT are shortened by 19.88%, 19.39%, and 20.83% compared to the control groups, respectively. As a result of poor goal orientation, complex paths were generated around the main and slave manipulators. The success rate of the G-RRT was only 19 and 18 times, respectively, while the GBI-RRT failed just once, and all other methods were executed successfully. It can be seen that the overall performance of the BPFPS-RRT is better than the control groups.

## 7. Limitations and Future Scope

The focus of this paper is on the path planning of dual manipulators in three-dimensional environments, and the proposed algorithm can also be applied to various types of robots, such as mobile cars, drones, etc. In practical applications in other intelligent fields, such as intelligent congestion control [36], only map information is required to plan the optimal path, and the BPFPS-RRT algorithm has demonstrated good path planning performance in this field by taking advantage of each improved strategy point. However, in practical applications in the field of robotics, its application challenges mainly lie in efficiency, real-time updates, and robot kinematics characteristics, which may result in some differences from the simulation experimental results. Therefore, in response to these issues and challenges, future research will focus on relevant practical application research by combining the characteristics of dual manipulators and practical application scenarios. The objective is to conduct experiments to optimize algorithms in order to improve the operational efficiency and reliability of path planning in practical applications.

## 8. Conclusions

In this paper, a path planning method with angle-selected bidirectional potential field probabilistic step size RRT is proposed. To balance the randomness and blindness of the algorithm, the goal bias angle and random values are used as the strategy for step size searches, the artificial potential field method is introduced, and the bidirectional goal bias features of searches and the concept of greedy path optimization are combined. By analyzing the performance of the algorithms, it was found that the search time of the BPFPS-RRT algorithm is reduced by 24.50%, 10.76%, and 36.39%, respectively, compared with the G-RRT, VT-RRT, and GBI-RRT algorithms, the path lengths are reduced by 13.38%, 13.03%, and 18.38%, respectively, and the performance of the improved strategy is verified by an ablation experiment. Therefore, the planning path and search time of the proposed algorithm are more advantageous than the control groups. For the obstacle avoidance problem in path planning, the proposed algorithm is applied to the main manipulator of the dual manipulator. The search time of the proposed algorithm is reduced by 23.53%, 15.45%, and 43.78%, respectively, relative to the control groups, and the path lengths are reduced by 19.35%, 18.83%, and 21.38%, respectively. On the slave manipulator of the dual manipulator, the search time of the proposed algorithm is reduced by 6.71%, 1.49%, and 46.88% relative to the control groups, respectively, and the path lengths are reduced by 19.88%, 19.39%, and 20.83%, respectively. The simulation results show that the dual manipulator successfully avoids the obstacles to reach the goal position, and the trajectory of each joint is smooth.

## Figures and Tables

**Table 1:** The types of path planning algorithms.

Types	Algorithms	Refs.	Advantages	Disadvantages	Reasons for Disadvantages
Based on search algorithms.	Dijkstra and A*, etc.	[6,7,8]	Find the optimal path at a reasonable time.	Large search space, high computational complexity, and poor real-time performance.	In complex environments, it faces a large amount of path search calculations.
Based on rules algorithms.	APF and variants, etc.	[9,10,11]	Fast path calculation and high real-time performance.	Local optima are prone to occur.	Neglecting the mutual constraints between robots.
Based on sampling algorithms.	PRM and variants, etc.	[13,14,15]	High efficiency and real-time performance.	Low reuse rate of roadmap and high memory consumption.	Generating a large number of candidate paths comes with relatively high computational costs.
	RRT and variants, etc.	[12,16,17,18,19,20,21,22]	Strong applicability, high real-time performance, high efficiency, and scalability.	There are many tree nodes and poor path quality.	Affected by random factors, the results may be unstable.

**Table 2:** DH parameters of the main manipulator.

Links	$\theta_{i}$	$d_{i}\;(m)$	$a_{i}\;(m)$	$\alpha_{i}$ $\;({}^{{}^{\circ}})$	$\mathrm{Range}\;({}^{{}^{\circ}})$
1	$\theta_{1}$	0	0	90	−160~160
2	$\theta_{2}$	0	$a_{2}$	0	−225~45
3	$\theta_{3}$	$d_{3}$	$a_{3}$	−90	−45~225
4	$\theta_{4}$	$d_{4}$	0	90	−110~170
5	$\theta_{5}$	0	0	−90	−100~100
6	$\theta_{6}$	0	0	0	−226~226

**Table 3:** Average experimental data of the four algorithms.

Algorithms	G-RRT	VT-RRT	GBI-RRT	BPFPS-RRT
Search time (s)	5.51	4.46	6.54	4.16
Path length (mm)	315.36	314.11	334.68	273.16
Tree nodes	44	41	68	35
Path nodes	31	23	34	13

**Table 4:** Average experimental data of the five algorithms.

Algorithms	RRT-Connect	A	A + B	A + B + C	BPFPS-RRT
Search time (s)	7.42	6.88	5.27	3.28	3.19
Path length (mm)	360.88	348.79	320.18	273.81	254.70
Tree nodes	88	85	64	38	35
Path nodes	38	36	33	17	10

**Table 5:** Motion parameters of the dual manipulator.

Objects	Motion Parameters
Main manipulator coordinate origin	(0, 0.5, 0)
Salve manipulator coordinate origin	(0, −0.5, 0)
$\mathrm{Initial}\;\mathrm{position}\;\mathrm{of}\;\mathrm{main}\;\mathrm{manipulator}/({}^{{}^{\circ}})$	[30, 135, 0, 180, 135, 150]
$\mathrm{Goal}\;\mathrm{position}\;\mathrm{of}\;\mathrm{main}\;\mathrm{manipulator}/({}^{{}^{\circ}})$	[135, 130, 15, 180, 150, 45]
$\mathrm{Initial}\;\mathrm{position}\;\mathrm{of}\;\mathrm{salve}\;\mathrm{manipulator}/({}^{{}^{\circ}})$	[315, 135, 15, 180, 140, 135]
$\mathrm{Goal}\;\mathrm{position}\;\mathrm{of}\;\mathrm{salve}\;\mathrm{manipulator}/({}^{{}^{\circ}})$	[210, 135, 0, 180, 135, −30]
Fixed step size δ/cm	0.1
Safety distance/cm	0.2
Sphere1	(0, 0, 0)
Sphere2	(−0.25, 0.25, 0)
Sphere3	(0.4, 0.2, 0.4)
Sphere4	(0, 0, 0.4)

**Table 6:** Average running data of the four algorithms.

Dual Manipulator	Main Manipulator				Salve Manipulator			
Algorithms	G-RRT	VT-RRT	GBI-RRT	BPFPS-RRT	G-RRT	VT-RRT	GBI-RRT	BPFPS-RRT
Average runtime (s)	2.72	2.46	3.7	2.08	2.83	2.68	4.97	2.64
Average path length (cm)	1.55	1.54	1.59	1.25	1.66	1.65	1.68	1.33
Success times	19	20	19	20	18	20	19	20

## Data Availability

Not applicable.

## Nomenclature

$d_{i}$	offset of the two adjacent links (m)
$a_{i}$	length of the links (m)
*D*	distance between the two manipulators (m)
x	x-direction (mm)
y	y-direction (mm)
z	z-direction (mm)
$\alpha_{i}$	torsion angle of the links (${}^{{}^{\circ}}$)
$\theta_{i}$	the angle between the two adjacent links (${}^{{}^{\circ}}$)
$R_{rot}\left(\cdot ,\cdot \right)$	rotational transformation matrix
$T_{trans}(\cdot ,\cdot )$	translational transformation matrix
c	cosine function
s	sine function
$S_{base}$	transformation matrix of the base coordinate system
$R_{ai}$,$R_{bj}$	the radii of the left and right manipulator link encirclement boxes (mm)
$O_{ai}$,$O_{bj}$	the center points of each link joint
$d_{min}$	the shortest distance between the two links (mm)
i,j	count
δ	the fixed step size (mm)
γ	a random value prior to expansion (mm)
*P_a_*	the threshold of high goal probability
$\delta_{1}$	randomly varying the step size for shortening (mm)
$\delta_{2}$	randomly varying the step size for increasing (mm)
Q	node
$Q_{rand1}$	the nearest neighbor random point of the starting point
$Q_{rand2}$	the nearest neighbor random point of the goal point
***D*_1_**	the vectors of $Q_{nearest}$ random point
***D*_2_**	the vectors of $Q_{nearest}$ goal point
θ	the angle between $D_{1}$ and $D_{2}$ (${}^{{}^{\circ}}$)
$S_{r}$	the probability step size base on angle selection (mm)
$k_{g}$	gravitational proportional coefficient
$k_{r}$	proportional coefficient of repulsion
$F_{g}$	gravitational field step size (mm)
$F_{r}$	repulsive field step size (mm)
F	the step size of the combined force (mm)
C	the set of nodes
*S*	starting node
*N*	the sequence of path nodes
G	goal point
$n_{i-1}$	the current node
v	retention point
